# Taquicardia Ventricular Não Sustentada e Cardiomiopatia Hipertrófica: Quando Considerá-la como Fator de Risco para Morte Súbita e Mortalidade Total?

**DOI:** 10.36660/abc.20250233

**Published:** 2025-05-29

**Authors:** Lucas Simonetto Faganello, Mauricio Pimentel

**Affiliations:** 1 Hospital de Clinicas de Porto Alegre Porto Alegre RS Brasil Hospital de Clinicas de Porto Alegre, Porto Alegre, RS – Brasil

**Keywords:** Cardiomiopatia Hipertrófica, Taquicardia Ventricular, Mortalidade

A cardiomiopatia hipertrófica (CMH) é uma causa importante de morte súbita cardíaca (MSC), particularmente em pacientes jovens.^1 , 2^ O implante de um cardioversor-desfibrilador implantável (CDI) em pacientes de alto risco é um aspecto fundamental do tratamento da CMH. No entanto, atualmente não há ensaios clínicos randomizados para orientar a seleção de pacientes candidatos ao implante de CDI. As recomendações são baseadas em coortes retrospectivas que identificaram a relação entre características clínicas e prognóstico. Os fatores de risco para MSC incluem histórico de síncope ou taquiarritmia ventricular, histórico familiar de morte súbita, espessamento da parede ventricular esquerda, disfunção sistólica, aneurisma apical, realce tardio na ressonância magnética e presença de taquicardia ventricular não sustentada (TVNS).^1^

A TVNS ocorre em 20-30% dos pacientes com CMH e é um preditor independente de MSC.^2 - 6^ No entanto, a força dessa evidência varia entre os estudos observacionais publicados, frequentemente devido a diferenças nas populações estudadas ou nos critérios diagnósticos. Pesquisas conduzidas em centros terciários indicam que a presença de TVNS está associada a um maior risco de MSC, enquanto estudos de centros não referenciados não identificaram uma associação estatisticamente significativa.^7^ Outro ponto de discussão são as características da TVNS que devem ser consideradas. Enquanto a diretriz americana dá maior ênfase a arritmias mais frequentes, mais longas e mais rápidas, a diretriz europeia argumenta que não há evidências robustas para apoiar isso.^8 , 9^ A diretriz brasileira considera a TVNS relevante quando é frequente (≥ 3), mais longa (≥ 10 batimentos) e mais rápida (≥ 200 batimentos por minuto).^10^

Esta edição dos Arquivos Brasileiros de Cardiologia apresenta um estudo de coorte brasileiro de pacientes com CMH acompanhados por 15 anos.^11^ Retrospectivamente, foram avaliados 763 pacientes, dos quais 8,3% possuíam marcapasso e 6,8% CDI. A forma obstrutiva da doença foi observada em 28,7% dos pacientes, e apenas 5,6% apresentaram espessura septal > 30 mm. Os autores definiram a presença de TVNS com base nos achados de um Holter de 24 horas indicando TVNS longa e rápida (duração > 10 batimentos e frequência cardíaca > 130 bpm) ou na ocorrência de pelo menos 3 episódios de TVNS com duração > 3 batimentos e frequência cardíaca > 120 bpm. A incidência de TVNS foi de 10%. Ao considerar apenas o critério de episódios longos e rápidos, a incidência foi de 1,4%. A presença de TVNS não demonstrou associação com variáveis clínicas como sexo, idade > 40 anos ou espessura do septo interventricular. A mortalidade por todas as causas ao longo do estudo foi significativamente maior no grupo de pacientes com TVNS (26,3% vs. 15,9%).

O estudo apresenta limitações típicas de uma coorte retrospectiva. A amostra de conveniência não controla a seleção de pacientes, e a coleta de dados de prontuários limita as informações disponíveis, tornando qualquer inferência estatística exploratória, conforme observado pelos autores. A prevalência de TVNS em pacientes com CMH apresenta variabilidade significativa entre os diferentes estudos observacionais publicados.^2 - 7^ Essa variabilidade está claramente relacionada à seleção de pacientes (pacientes com doença mais grave tendem a apresentar mais arritmias em comparação aos demais), aos critérios para definição de TVNS e à duração do monitoramento. No estudo, a média de idade dos pacientes foi de 52,6 anos, maior do que na maioria dos estudos prognósticos sobre TVNS em CMH. Uma limitação importante do estudo é que ele avaliou apenas a mortalidade total e não abordou especificamente a MSC. Essas informações são altamente relevantes para a tomada de decisão clínica em relação ao implante de CDI. Mesmo com suas limitações, o estudo é bastante relevante para avaliar uma coorte nacional em uma doença com componente genético bem definido. A aplicação de estratégias de estratificação de risco derivadas de estudos internacionais em pacientes brasileiros pode não produzir os mesmos resultados.^12 , 13^

A estratificação de risco para MSC em pacientes com CMH permanece desafiadora. A disponibilidade de calculadoras de risco ou fluxogramas claros, como os da diretriz brasileira, facilita o julgamento clínico, mas não define individualmente o prognóstico dos pacientes. A presença e a morfologia da TVNS, bem como sua relação com outros fatores de risco clássicos para MSC, precisam ser melhor avaliadas em estudos multicêntricos maiores para determinar quais pacientes realmente se beneficiam do implante de CDI.
