# Supplementary material for: Taquicardia Ventricular Não Sustentada e Cardiomiopatia Hipertrófica: Quando Considerá-la como Fator de Risco para Morte Súbita e Mortalidade Total?
Source: Arq Bras Cardiol. 2025 May 29;122(5):e20250233. [Article in Portuguese] doi: 10.36660/abc.20250233 (PMC12212382; doi:10.36660/abc.20250233)
Supplement: Translation

# Translation: Non-sustained Ventricular Tachycardia and Hypertrophic Cardiomyopathy: When to Consider it as a Risk Factor for Sudden Death and Total Mortality?

**DOI:** 10.36660/abc.20250233i

**Authors:** Lucas Simonetto Faganello, Mauricio Pimentel

**Affiliations:** 1 Hospital de Clinicas de Porto Alegre Porto Alegre RS Brazil Hospital de Clinicas de Porto Alegre, Porto Alegre, RS – Brazil

**Keywords:** Hypertrophic Cardiomyopathy, Ventricular Tachycardia, Mortality

Hypertrophic cardiomyopathy (HCM) is an important cause of sudden cardiac death (SCD), particularly in young patients.^1 , 2^The implantation of an implantable cardioverter-defibrillator (ICD) in high-risk patients is a pivotal aspect of HCM treatment. However, there are currently no randomized clinical trials to guide the selection of patients who are candidates for ICD implantation. Recommendations are based on retrospective cohorts that have identified the relationship between clinical characteristics and prognosis. Risk factors for SCD include history of syncope or ventricular tachyarrhythmia, family history of sudden death, left ventricular wall thickness, systolic dysfunction, apical aneurysm, late enhancement on magnetic resonance imaging, and the presence of non-sustained ventricular tachycardia (NSVT).^1^

NSVT occurs in 20-30% of patients with HCM and is an independent predictor of SCD.^2 - 6^However, the strength of this evidence varies among published observational studies, often due to differences in the studied populations or diagnostic criteria. Research conducted in tertiary centers indicates that the presence of NSVT is associated with a higher risk of SCD, while studies from non-reference centers have not identified a statistically significant association.^7^Another point of discussion is the characteristics of NSVT that should be considered. While the American guideline places greater emphasis on more frequent, longer, and faster arrhythmias, the European guideline argues that there is no robust evidence to support this.^8 , 9^The Brazilian guideline considers NSVT relevant when it is frequent (≥ 3), longer (≥ 10 beats), and faster (≥ 200 beatsper minute).^10^

This edition of the *Arquivos Brasileiros de Cardiologia* presents a Brazilian cohort study of patients with HCM followed over 15 years.^11^Retrospectively, 763 patients were evaluated, of which 8.3% had pacemakers and 6.8% had ICDs. The obstructive form of the disease was observed in 28.7% of the patients, and only 5.6% presented with septal thickness > 30 mm. The authors defined the presence of NSVT based on findings from a 24-hour Holter monitor indicating long and fast NSVT (duration ≥ 10 beats and heart rate ≥ 130 bpm) or the occurrence of at least 3 episodes of NSVT with a duration ≥ 3 beats and heart rate ≥ 120 bpm. The incidence of NSVT was 10%. When considering only the criterion of long and fast episodes, the incidence was 1.4%. The presence of NSVT did not show an association with clinical variables such as sex, age > 40 years, or interventricular septal thickness. All-cause mortality throughout the study was significantly higher in the group of patients with NSVT (26.3% vs. 15.9%).

The study presents typical limitations of a retrospective cohort. The convenience sample does not control patient selection, and data collection from records limits the available information, making any statistical inference exploratory, as noted by the authors. The prevalence of NSVT in patients with HCM shows significant variability across different published observational studies.^2 - 7^This variability is clearly related to patient selection (patients with more severe disease tend to have more arrhythmias compared to others), the criteria for defining NSVT, and the duration of monitoring. In the study, the mean age of patients was 52.6 years, which is higher than in most prognostic studies on NSVT in HCM. A major limitation of the study is that it evaluated only total mortality and did not specifically address SCD. This information is highly relevant for clinical decision-making regarding ICD implantation. Even with its limitations, the study is quite relevant for evaluating a national cohort in a disease that has a well-defined genetic component. The application of risk stratification strategies derived from international studies to Brazilian patients may not yield the same results.^12 , 13^

Risk stratification for SCD in patients with HCM remains challenging. The availability of risk calculators or clear flowcharts, such as those in the Brazilian guideline, facilitates clinical judgment but does not individually define patients’ prognosis. The presence and morphology of NSVT, as well as its relationship with other classical risk factors for SCD, need to be better evaluated in larger multicentric studies to determine which patients truly benefit from ICD implantation.
